# SNPs in microRNA seed region and impact of miR-375 in concurrent regulation of multiple lipid accumulation-related genes

**DOI:** 10.1038/s41598-024-61673-4

**Published:** 2024-05-13

**Authors:** Jiyeon Lee, Inpyo Hong, Chanwoo Lee, Daehyun Kim, Sunghak Kim, Yoonseok Lee

**Affiliations:** 1 School of Biotechnology, Hankyong National University, Anseong, Gyeonggi-do South Korea; 2 Nuonbio Inc., 906, A, 302 Galmachi-ro, Jungwon-gu, Seongnam-si, South Korea; 3 Department of Animal Science, Chonnam National University, Gwangju, South Korea; 4 Center for Genetic Information, Hankyong National University, Anseong, Gyeonggi-do South Korea

**Keywords:** Bovine, miR-375, SNP, GPAM, Lipid metabolism, Adipocyte, Predictive markers, Animal breeding

## Abstract

Bovine intramuscular fat (IMF), commonly referred to as marbling, is regulated by lipid metabolism, which includes adipogenesis, lipogenesis, glycerolipid synthesis, and lipolysis. In recent years, breeding researchers have identified single nucleotide polymorphisms (SNPs) as useful marker-assisted selection tools for improving marbling scores in national breeding programs. These included causal SNPs that induce phenotypic variation. MicroRNAs (miRNAs) are small highly conserved non-coding RNA molecules that bind to multiple non-coding regions. They are involved in post-transcriptional regulation. Multiple miRNAs may regulate a given target. Previously, three SNPs in the *GPAM* 3ʹ UTR and four miRNAs were identified through in silico assays. The aim of this study is to verify the binding ability of the four miRNAs to the SNPs within the 3ʹUTR of *GPAM*, and to identify the regulatory function of miR-375 in the expression of genes related to lipid metabolism in mammalian adipocytes. It was verified that the four miRNAs bind to the *GPAM* 3ʹUTR, and identified that the miR-375 sequence is highly conserved. Furthermore, it was founded that miR-375 upregulated the *GPAM* gene, *C/EBPα*, *PPARγ* and lipid metabolism-related genes and promoted lipid droplet accumulation in 3T3-L1 cells. In conclusion, these results suggest that miR-375 is a multifunctional regulator of multiple lipid metabolism-related genes and may aid in obesity research as a biomarker.

## Introduction

Intramuscular fat (IMF) deposition plays a crucial role in meat quality and is a key factor that influences consumer preferences in the Korean native cattle (Hanwoo) industry. IMF is commonly known as marbling and refers to the fat deposited between muscle fibers in cattle^1^. The function of IMF differs from the physiological functions of external or subcutaneous fat. The IMF level is determined by the regulation of lipid metabolism, which includes adipogenesis, lipogenesis, glycerolipid synthesis, and lipolysis^2^. Genetically, Hanwoo cattle can potentially express IMF deposition similar to that of Wagyu cattle, resulting in more marbling than that in European breeds^1^. Several breeding studies reported that a whole-genome bovine single nucleotide polymorphism (SNP) panel containing significant SNPs such as BTB-01280026 for Hanwoo cattle could be used to improve meat quality in national breeding programs^3–5^. Causal SNPs are located in non-coding regions and can affect gene expression through transcriptional or post-transcriptional regulation^6^ by altering the interactions with RNA or other biomolecules to produce phenotypic variations^7^. These SNPs modulate secondary RNA structures and influence gene expression via post-transcriptional regulation^8^. Thus, the accuracy of Hanwoo genome selection has been significantly improved by using a high-density causal SNP panel^9^.

Regulation of gene expression via transcription factors and small regulatory factors, such as SNP, directly influences protein production and phenotypic variation^7^. The genome primarily encodes noncoding RNAs (ncRNAs), with only approximately 2% encoding proteins. Thus, ncRNAs play essential roles in various biological processes^10^. MicroRNAs (miRNAs) are small ncRNAs that are approximately 22 nucleotides long and act as post-transcriptional regulators^11^. Additionally, miRNAs regulate approximately 50% of the protein-coding genes in mammals^12^. The 6 nt seed region in the 5ʹ end of the miRNA recognizes the target mRNA, and owing to the preferential conservation of miRNA target sites over other regions of the 3ʹ untranslated region (UTR), these sites are particularly susceptible to mutations, structural variations, and gene conversion^13,14^. A single gene may be regulated by multiple miRNAs. Conversely, a single miRNA can target multiple genes and alter the expression of approximately 400 genes on average. More than 45,000 miRNA target sites are estimated to be conserved in the 3ʹ UTR of miRNAs at background levels^14^. Additionally, deletion of a single miRNA may cause phenotypic changes because of the complex and highly regulated network of interactions between miRNAs and mRNAs within the genetic network^15,16^. In contrast to previously reported one-to-one relationship between miRNAs and mRNAs^17^, miRNAs have been recently shown to simultaneously regulate multiple biological pathways and genes. For example, miR-335 upregulates several adipocyte differentiation genes in the mouse liver and white adipose tissue^18^. miR-185 downregulates the mRNA expression of multiple lipid metabolism-related genes in human hepatocytes and promotes the insulin signaling pathway in mice, thereby improving lipid accumulation^19^. miR-27b regulates several key lipid metabolism-related genes in human hepatocytes^20^. miR-224 regulates intramuscular fat by downregulating fat-formation-related genes in bovine adipocytes^21^. The functions of miRNAs in regulating multiple lipid metabolism-related genes have been extensively investigated in mice and humans, and numerous studies have focused on bovine intramuscular fat-related miRNAs^22–24^. SNPs influence miRNA-mediated gene regulation. SNPs in the miRNA target site of the mRNA 3ʹ UTR are polymorphisms in miRNAs and their target sites (poly-miRTSs) and affect miRNA activity^25^. SNPs were first identified as causes of diseases in humans, and the interactions between miRNAs and SNPs have been recently studied in livestock. Li et al. reported that the binding of bta-miR-744 changed depending on the type of SNP within the 3ʹ UTR of the bovine FADS2 gene^26^. Ju et al*.* reported that the miRNA–mRNA interaction changed depending on the type of SNP within the 3ʹ UTR of the bovine *NCF4* gene^27^. However, the interactions between bovine IMF-related SNPs and miRNAs are poorly understood.

Several studies have reported that the upregulation of the mRNA expression of glycerolipid synthesis-related genes increased IMF deposition^28^. The glycerol-3-phosphate acyltransferase 1 (*GPAT1*) gene catalyzes the initial and concomitant steps of glycerolipid synthesis; this is important because glycerolipid synthesis correlates highly with IMF deposition in Hanwoo^29^. The glycerol-3-phosphate acyltransferase, mitochondrial (*GPAM*) gene, also known as *GPAT1*, has a longer 3ʹ UTR than that of other bovine genes. The SNP in the long 3ʹ UTR affects the miRNA binding structure, which is important because it interferes with gene expression at the post-transcriptional level^30,31^. In silico assays have predicted the binding of three SNPs (g.54853A>G, g.55441A>G, and g.55930C>T) in the *GPAM* 3ʹ UTR of Hanwoo with four miRNAs (bta-miR-2418, bta-miR-375, bta-miR-2479, bta-miR-2468). Thus, this study aimed to verify the binding ability of four miRNAs to each SNP combination type of the *GPAM* 3ʹ UTR and investigate the effect elicited on the phenotype by miRNA-mediated regulation of the expression of several lipid metabolism-related genes in adipocytes.

## Results

### Direct binding of miRNAs to the 3ʹUTR of bovine *GPAM*

**Figure 1:**
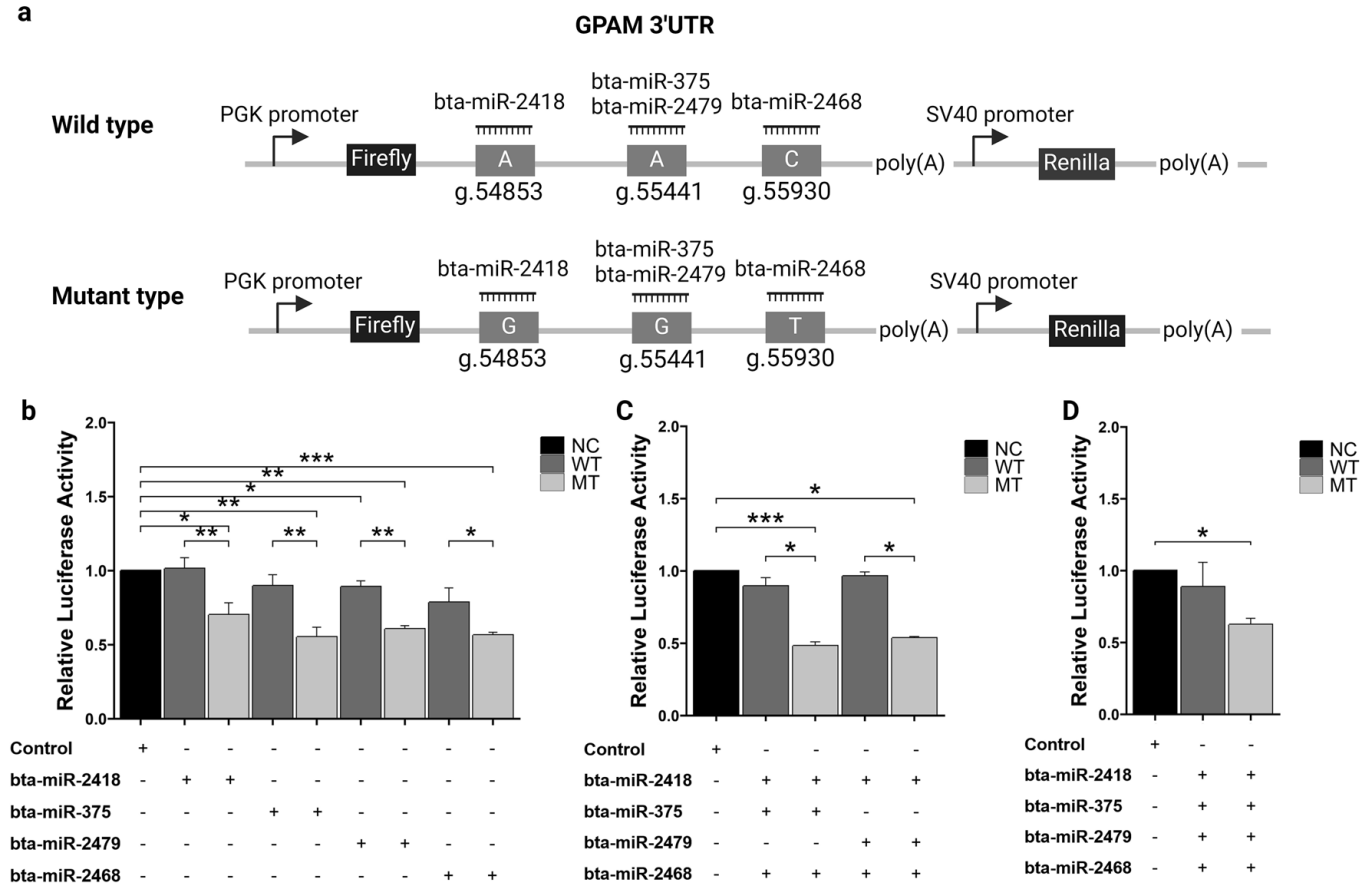
Relative luciferase activity of single, triple, and combination microRNA binding to wild-type and mutants within the 3ʹ UTR of *GPAM*. (**a**) Schematic diagram of the luciferase reporter vectors with two allele types of the *GPAM* 3ʹ UTR containing three SNPs (g.54853, g.55441, and g.55930). Two types of recombinant DNA were constructed by inserting the *GPAM* mutant and wild-type alleles into the pmirGLO vector, and the GPAM SNPs are located within the target site of bta-miR-2418, bta-miR-375, bta-miR-2479, and bta-miR-2468. (**b**–**d**) Relative luciferase activity according to the SNP combination type. The panels show the results of transfection of single, triple, and quadruple combinations of miRNAs. Luciferase activity was measured 48 h after transfection of miRNAs into HEK293T cells, and the firefly luciferase activity was normalized to that of Renilla luciferase activity. Each miRNA binds directly to the *GPAM* 3ʹ UTR and the relative luciferase activity is significantly reduced in the presence of the mutant-type allele but not of the wild-type allele. Binding affinity between the mutant-type and the wild-type alleles differed significantly. NC, negative control; WT, wild-type; MT, mutant-type. Data are shown as the mean ± SD (n = 3). p-values were calculated using Student’s t-test. *p < 0.05; **p < 0.01; ***p < 0.001.

**Figure 2:**
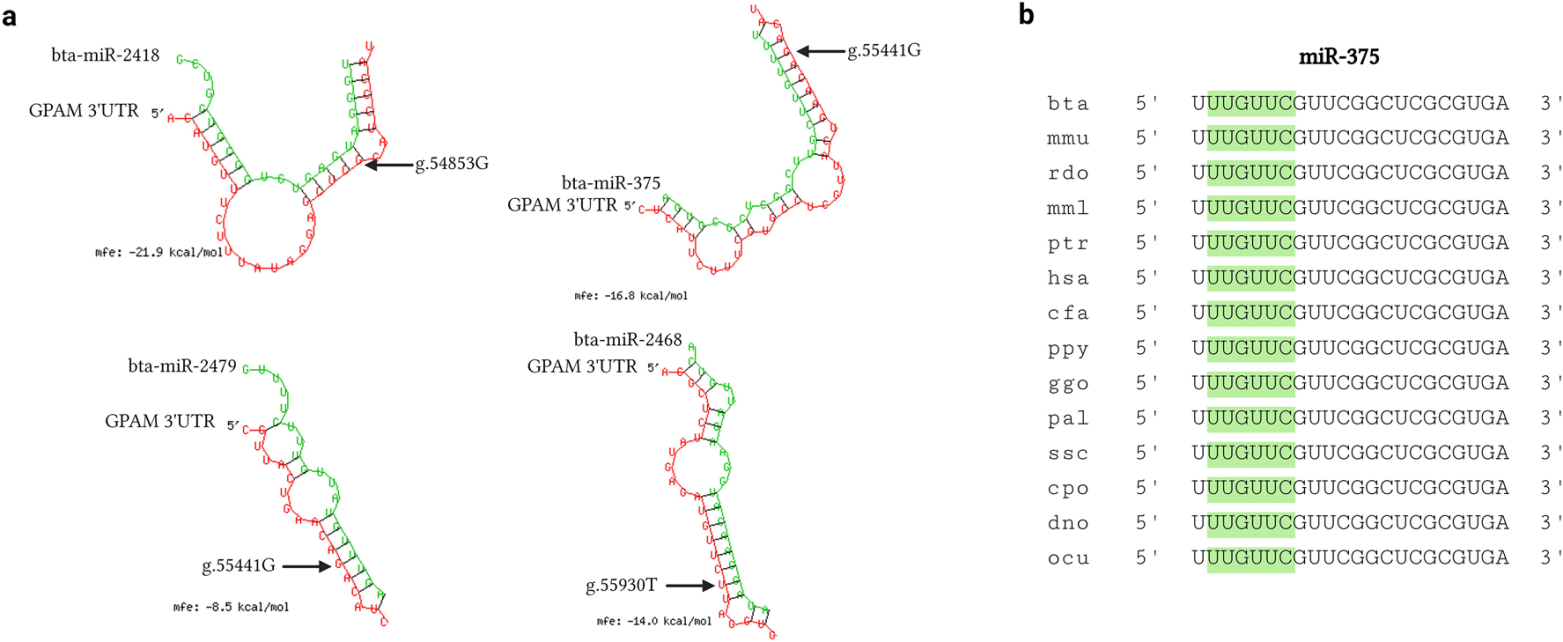
*GPAM* 3ʹ UTR SNPs as target of bta-miR-2418, bta-miR-375, bta-miR-2479, and bta-miR-2468. (**a**) Schematic diagram of the interaction between SNPs g.54853A>G, g.55441A>G, and g.55930C>T and bta-miR-2418, bta-miR-375, bta-miR-2479, and bta-miR-2468 analyzed using RNAhybrid. The panel shows the structure predicted to bind to the SNP of bta-miR-2418, bta-miR-375, bta-miR-2479, and bta-miR-2468. The SNPs are located in the miRNA seed region of the 5ʹ end from 2–7 nt. The SNP in the seed region is shown in red. The position of the SNPs in the *GPAM* 3ʹ UTR is indicated by an arrow; and the miRNA and mRNA are shown as the green and red strands, respectively. (**b**) Conservation of miR-375 mature sequences among various species. miRNA mature sequences were searched in miRBase v.22.1 and MirGeneDB 2.1. The miR-375 sequence is widely conserved among different species, and bta-miR -375 sequence perfectly matched with that of 14 species, including mmu-miR-375.

The SNPs were discovered according to a previous study^32^, SNP g.54853A>G, g.55441A>G, and g.55930C>T were identified and found to have a significant correlation with marbling scores in the Hanwoo cattle population. In addition, the AAC and GGT haplotypes were most frequently observed within the different combinations of the three SNPs. Furthermore, computational simulations and online miRNA database analyses identified four miRNAs with the potential to bind to the SNPs of *GPAM* 3ʹ UTR, suggesting different binding affinities for each SNP combination type^32^. The binding affinities of these miRNAs to both wild-type (g.54853A, g.55441A, and g.55930C) and mutant-type (g.54853G, g.55441G, and g.55930T) alleles were validated by constructing recombinant DNA using the pmirGLO vector comprising the wild and mutant-type alleles of the 3ʹ UTR fragment (Fig. 1a). The constructed recombinant DNA containing the SNPs was validated by sequencing (Supplementary Fig. S1b). These constructs were co-transfected with a single or combination of miRNA mimics (bta-miR-2418, bta-miR-375, bta-miR-2479, and bta-miR-2468) or negative control mimics into HEK293T cells. Then, luciferase activity was quantified after 48 h. Figure 1 illustrates the different luciferase activities corresponding to one and combinations of three and four miRNAs that bind to both allele types. Notably, co-transfection with a single miRNA led to reduced luciferase activity in mutant DNA but not in wild-type DNA, except for bta-miR-2479 (Fig. 1b). A similar reduction in luciferase activity was observed in mutant DNA with a combination of three and four miRNAs (Fig. 1c,d). The individual miRNAs consistently reduced luciferase activity in the *GPAM* 3ʹ UTR mutant type in all combinations, indicating direct binding of miRNAs to the 3ʹ UTR of *GPAM*. Furthermore, a significant difference in luciferase activity was observed between the wild-type and mutant alleles of the *GPAM* 3ʹ UTR. These results demonstrate that bta-miR-2418, bta-miR-375, bta-miR-2479, and bta-miR-2468 regulate the expression of the *GPAM* gene by directly binding to its 3ʹ UTR. Additionally, the binding affinity between each SNP combination type was significantly different in one and three, four miRNA combinations as shown in Fig. 1b–d. The SNPs within the *GPAM* 3ʹ UTR located in the miRNA seed region, which includes the second to the seventh nucleotides at the 5ʹ end, altered the miRNA–mRNA binding affinity. Consequently, these results confirm that miRNA–mRNA interactions are regulated by specific *GPAM* SNP types within this seed region (Fig. 2a), resulting in a varying in miRNA–mRNA binding affinities.

### Conservation of miRNA seed sequences across species

The aim of study is to determine if the four miRNAs (bta-miR-2418, bta-miR-375, bta-miR-2479, and bta-miR-2468) are evolutionarily conserved across species. miRNA conservation analysis was performed using miRBase and MirGeneDB, which validated their conservation within the same miRNA family and selected miRNAs with identical mature sequences.

The miRNA families bta-miR-2418, bta-miR-2479, and bta-miR-2468 were not detected in the results, implying that these miRNAs were uniquely expressed in *Bos taurus* species (Table 1). 70 families of miR-375 were identified, which are known to be evolutionarily conserved miRNAs. However, only 14 species possessed miR-375 with mature sequences identical to those of bta-miR-375. Moreover, the bta-miR-375 sequence was highly conserved among mammalian species, including cattle, mice, dogs, and humans, highlighting its essential role in mammals in general (Fig. 2b).

**Table 1:** Details of four miRNAs: seed type, binding region of *GPAM* transcripts, seed region (underlined), site of *GPAM* SNP (bold), and the number of species with conserved sequences.

miRNA	Seed type	Binding region	Target SNP	miRNA sequence (5ʹ→3ʹ)	Number of conserved species
bta-miR-2418	7mer-A1	4159–4169	g.54853 A>G	UGGGA***U***GAGUGUGGCGUGGUCG	1
bta-miR-375	7mer-m8	4747–4753	g.55441 A>G	U***U***UGUUCGUUCGGCUCGCGUGA	14
bta-miR-2479	7mer-A1	4749–4754	g.55441 A>G	AGU***U***UGUAUUGUUUCUUUUG	1
bta-miR-2468	7mer-m8	5235–5243	g.55930 C>T	AUA***G***GAACAUGGAAGAUUGUCA	1

Table 2 shows the regulatory effects of the binding of miR-375 to lipid metabolism-associated genes on gene expression levels in the four species. The 3T3-L1 cell line was used in this study as the experimental model owing to the significant homology between the cattle bta-miR-375 and mouse mmu-miR-375 sequences. Furthermore, homology analysis of the bovine and mouse *GPAM* genes revealed that mRNA homology was 84%, protein homology was 91%, and the 3ʹUTR binding site of miR-375 was 80% homologous between bovine and mouse.

**Table 2:** Regulation of gene expression by miR-375 in different species with corresponding target genes and their role.

Species	Target	Regulation	Function of gene
*Mus musculus*^48^	*CEBPα*	Up	Transcription factor that directly promotes expression of the adipogenic genes
	*PPARγ*	Up	Cooperative induction of adipogenesis with CEBPα
	*FABP4*	Up	Fatty acid uptake and transport for storage as triglycerides in lipid droplets
*Homo sapiens*^49,51^	*ADIPOQ*	Down	Adiponectin hormone, regulates blood glucose levels and promotes the degradation of fatty acids
	*ADIPOR2*	Down	Receptors of adiponectin secreted by adipose tissue
*Rattus norgevicus*^50, 52^	*GLUT4*	Up	Insulin-stimulated glucose uptake in adipocytes
	*LEP*	Down	Suppression of lipogenesis, energy balance in response to changes in fat stores
	*PDK1*	Down	Kinase that plays a critical role in the regulation of glucose and lipid metabolism
*Sus scrofa*^56^	*KLF2*	Up	Inhibition on PPARγ expression through direct binding to the *PPARγ* promoter

### miR-375-regulated gene expression in adipocytes

miR-375 regulates the expression of lipid metabolism-associated genes and influences differentiation in various cell types (Table 2). The objective of this study was to validate the regulatory effects of miR-375 on the expression of previously reported genes with a focus on lipid metabolism in different cell types and investigate the regulatory role of miR-375 in mouse *GPAM*. To confirm the transfection efficiency, miR-375 was transfected into 3T3-L1 cells, and the efficiency of transfection was confirmed using fluorescence microscopy (Supplementary Fig. S2).

Subsequently, we quantified the mRNA expression levels of *GPAM*, *GLUT4*, *CEBPα*, *PPARγ*, *ADIPOR2*, *ADIPOQ*, *PDK1*, *FABP4*, *KLF2*, and *LEP* genes using qRT-PCR.

**Figure 3:**
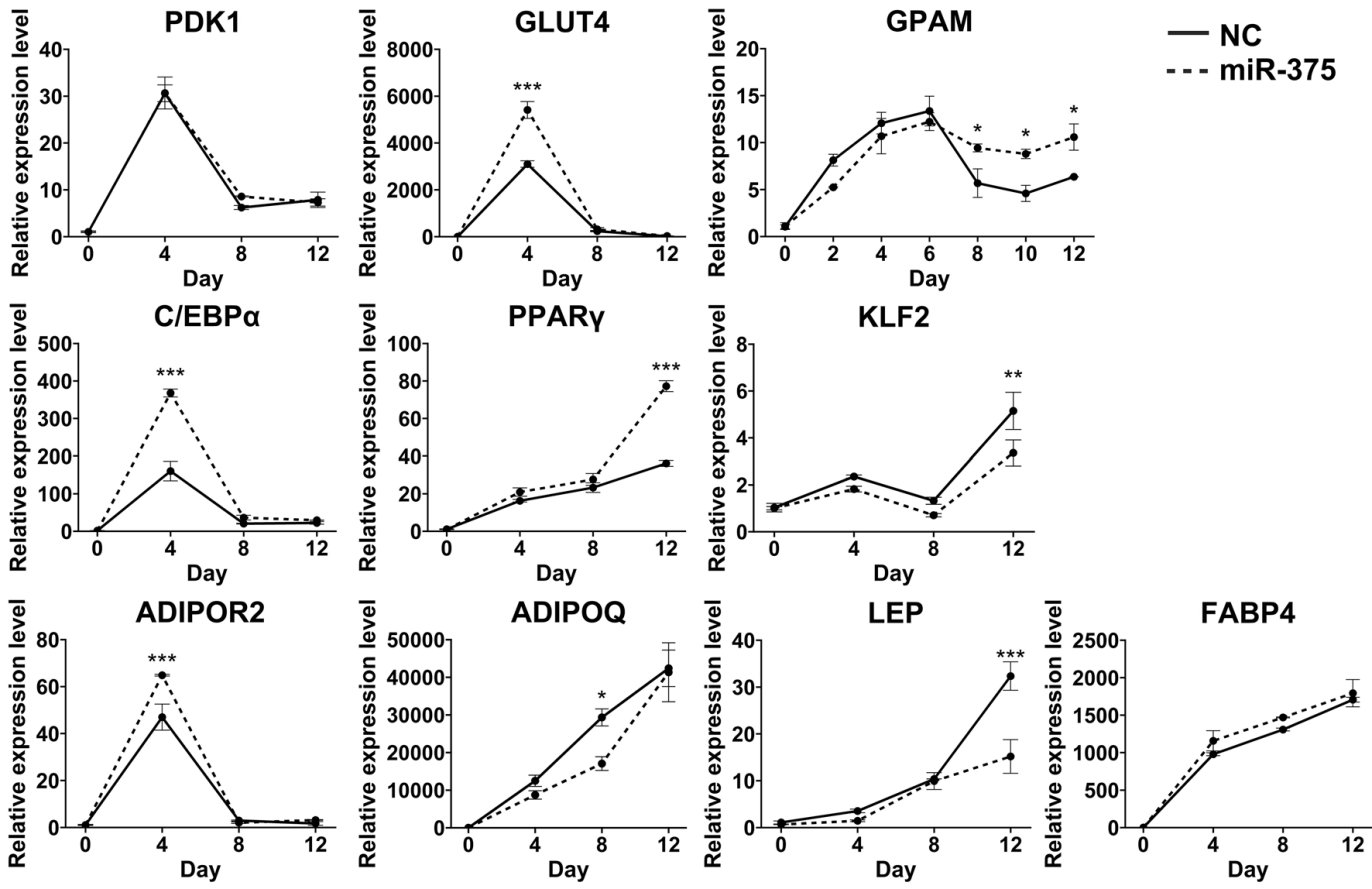
Relative mRNA expression levels of lipid metabolism-related genes during adipocyte differentiation. After transfection with miR-375 mimic or negative control (NC) mimic, qRT-PCR was performed to monitor changes in mRNA expression of lipid metabolism-related genes. Changes in mRNA expression levels were monitored every 2 days for *GPAM* gene and every 4 days for lipid metabolism-related genes. The mRNA expression level of the *GPAM* gene on days 8, 10, and 12 of differentiation was upregulated by miR-375 compared with that of the NC. The mRNA expression levels of the *GLUT4*, *CEBPα*, and *ADIPOR2* genes were upregulated on day 4 of differentiation, and the *PPARγ* gene was upregulated on day 12 of differentiation. *LEP* and *KLF2* mRNA expression levels were downregulated on day 12 and *ADIPOQ* mRNA expression was downregulated on day 8 of differentiation. *PDK1* and *FABP4* gene expression levels did not differ significantly. Data were normalized to relevant housekeeping genes and are shown as the mean ± SD (n = 3). mRNA expression was analyzed using two-way analysis of variance (ANOVA) with a threshold of *p < 0.05, **p < 0.01, ***p < 0.001.

Figure 3 illustrates the regulation of the expression of 10 lipid metabolism-associated genes in 3T3-L1 adipocytes on days 0, 2, 4, 6, 8, 10, and 12. As shown in Fig. 3, the relative expression levels of eight genes (*GLUT4*, *GPAM*, *CEBPα*, *PPARγ*, *KLF2*, *ADIPOR2*, *ADIPOQ*, and *LEP*) was significantly different between groups when transfecting miR-375 into 3T3-L1 adipocytes.

The relative expression level of the mouse *GPAM* gene gradually increased in both groups on days 0, 2, 4, and 6. However, the increase was not significantly different between groups and was lower in the miR-375-transfected group than in the negative control group. In contrast, gene expression significantly increased on days 8, 10, and 12 and was higher in the miR-375-transfected group than in the negative control group. The expression level of *GLUT4*, *CEBPα*, and *ADIPOR2* genes in 3T3-L1 adipocytes was significantly different between groups on day 4 and was higher in the miR-375-transfected group than in the negative control group.

Furthermore, on day 12, *PPARγ*, *KLF2*, and *LEP* expression in the 3T3-L1 adipocytes was significantly higher in the miR-375-transfected group than in the negative control group. Conversely, *ADIPOQ* expression was significantly lower in the miR-375-transfected group than in the negative control group.

### miR-375 induced enhanced lipid accumulation

**Figure 4:**
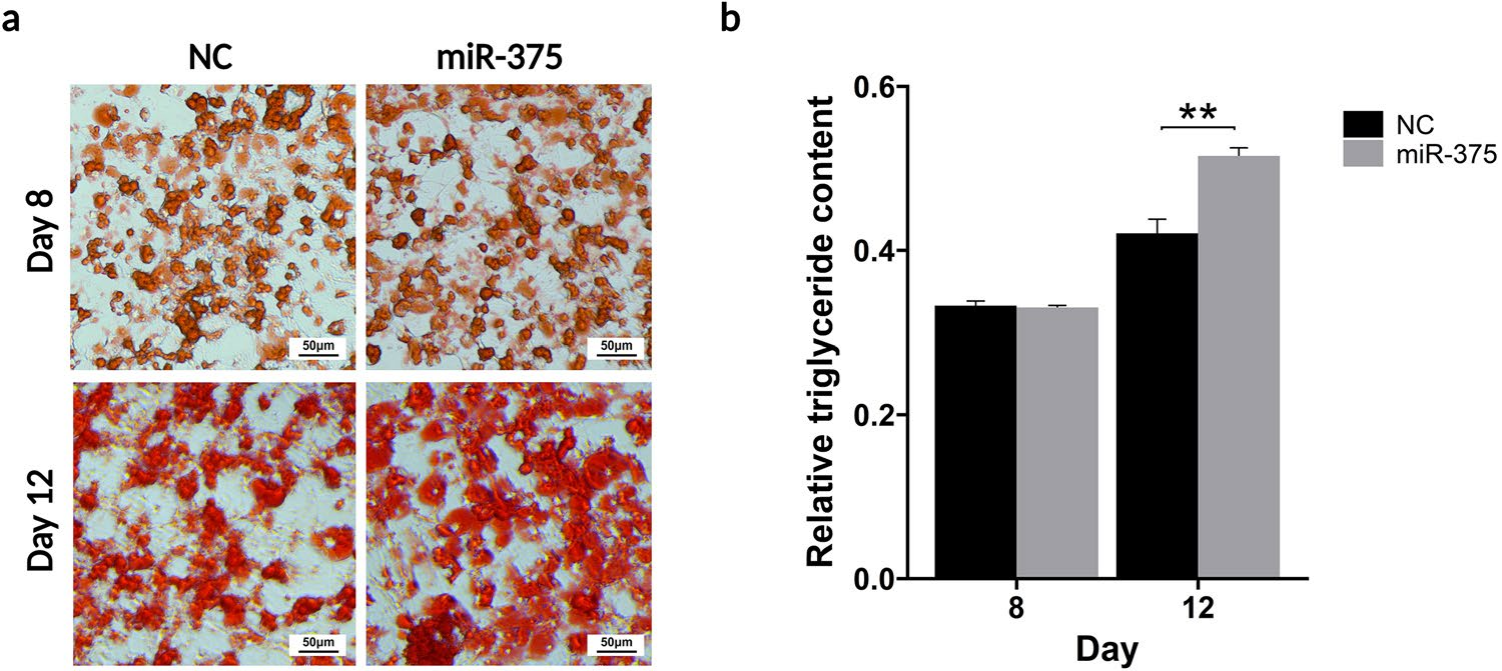
Lipid droplet deposition in 3T3-L1 cells after differentiation. (**a**) Oil Red O staining of negative control-treated and miR-375-treated 3T3-L1 adipocytes. The differentiation of 3T3-L1 preadipocytes to adipocytes was performed for 8 and 12 days. Intracellular lipid accumulation was measured using Oil Red O staining. Scale bar = 50 μm. (**b**) The lipid droplets in the adipocytes were quantified by measuring the absorbance at 510 nm. miR-375 increased lipid droplet deposition. The data are presented as the mean ± SD (n = 3). p-values were calculated using the Student’s t-test. **p < 0.01.

To investigate the influence of miR-375 on adipocyte differentiation and lipid accumulation, lipid droplets were analyzed using microscopy and quantified the triglyceride concentration in 3T3-L1 cells. miR-375 mimics were transfected into 3T3-L1 cells every 3 days, with differentiation continuing until day 12. As shown in Fig. 4a, 3T3-L1 pre-adipocyte differentiation and triglyceride accumulation were evaluated using Oil Red O staining on days 8 and 12. On day 8, total lipid accumulation did not significantly differ between the miR-375-transfected and negative control groups. Notably, total lipid accumulation significantly increased in the miR-375-transfected group compared with that in the negative control group on day 12 of 3T3-L1 differentiation (Fig. 4b). These results suggest that miR-375 enhances lipid accumulation in adipocytes during late differentiation, implying that miR-375 plays a significant role in increasing lipid accumulation in 3T3-L1 adipocytes by concurrently regulating the expression of multiple genes.

## Discussion

The aim of this study is to validate the binding affinity between mRNA 3ʹUTR SNP and miRNAs using a gene reporter assay and identify regulatory function of miRNA that influence IMF deposition in animal adipocytes. In our previous study, we identified three candidate functional SNPs within the *GPAM* gene that influence IMF deposition in Hanwoo. The regulatory relationship of these SNPs targeted by the four miRNAs was confirmed through computational analysis. Based on previous results, it has been verified that these four miRNAs (bta-miR-2418, bta-miR-375, bta-miR-2479, and bta-miR-2468) bind directly to the 3ʹ UTR of the bovine *GPAM*. Moreover, the results of the present study revealed a significant difference in binding affinity between the wild-type (g.54853A, g.55441A, and g.55930C) and mutant alleles (g.54853G, g.55441G, and g.55930T) in the miRNA seed region.

Glycerolipid synthesis is a metabolic pathway that converted glucose to glycerol-3-phosphate (G3P) to produce triglycerides. This process is regulated by glycerol-3-phosphate acyltransferases (GPATs), 1-acylglycerol-3-phosphate acyltransferases (AGPATs), lipin phosphatidic acid phosphatase (LIPIN), and diacylglycerol acyltransferases (DGAT). This process is initiated by the acylation of G3P by acyl-CoA (facilitated by *GPAM*), subsequently forming lysophosphatidic acid (LPA). Phosphatidic acid (PA), which is produced by the acylation of LPA, is dephosphorylated to form diacylglycerol (DAG), and DGAT adds fatty acids to DAG to form triglycerides^33^. In this pathway, *GPAM* catalyzes the initial step and is often targeted by miRNAs owing to its extended 3ʹ UTR^29^. Therefore, the miRNA regulatory activity of this enzyme needs to be investigated. Several studies have identified miRNAs that regulate *GPAM*. According to these results, miR-223 regulates IMF deposition by negatively targeting *GPAM* in chickens^34^. Additionally, miR-33b-5p interacts with musculin antisense RNA 1 (MSC-AS1), which is a long non-coding RNA, and directly targets *GPAM* in humans^35^. miR-494-3p down-regulates the basic helix-loop-helix ARNT-like protein 1 (BMAL1) gene its involvement in aberrant lipid metabolism, thereby enhancing GPAM-mediated lipid biosynthesis in hepatocellular carcinoma (HCC) cells^36^. miR-27b influences several key lipid metabolism genes, including *GPAM*, in the human hepatocyte cell line (Huh7)^20^. miR-29d-3p reduces triglyceride levels by inhibiting *GPAM* expression in bovine mammary epithelial cells (BMECs)^37^.

This study confirmed that bta-miR-2418, bta-miR-375, bta-miR-2479, and bta-miR-2468 have binding sites for *GPAM*, which could potentially regulate gene expression in Hanwoo cattle. Notably, we verified binding affinity varies depending on the *GPAM* SNP combination type, suggesting that SNPs present in the *GPAM* 3ʹ UTR may induce phenotypic variation upon interaction with miRNA. SNPs act as genetic markers for genomic selection in livestock because of their ability to induce phenotypic changes via single base substitutions^38^. SNPs occur in both coding and non-coding regions of the genome. Although SNPs within coding regions influence the protein structure through amino acid alterations^39^, most functional SNPs are located in non-coding regions and affect transcriptional regulation and post-transcriptional gene expression^6^. Post-transcriptional regulatory factors, such as miRNAs, modulate the translation of adipogenic genes^40^. Therefore, the three identified SNPs (g.54853A>G, g.55441A>G, g.55930C>T) and four miRNAs (bta-miR-2418, bta-miR-375, bta-miR-2479, and bta-miR-2468) could potentially be used as functional genetic markers for Hanwoo cattle genomic selection.

**Figure 5:**
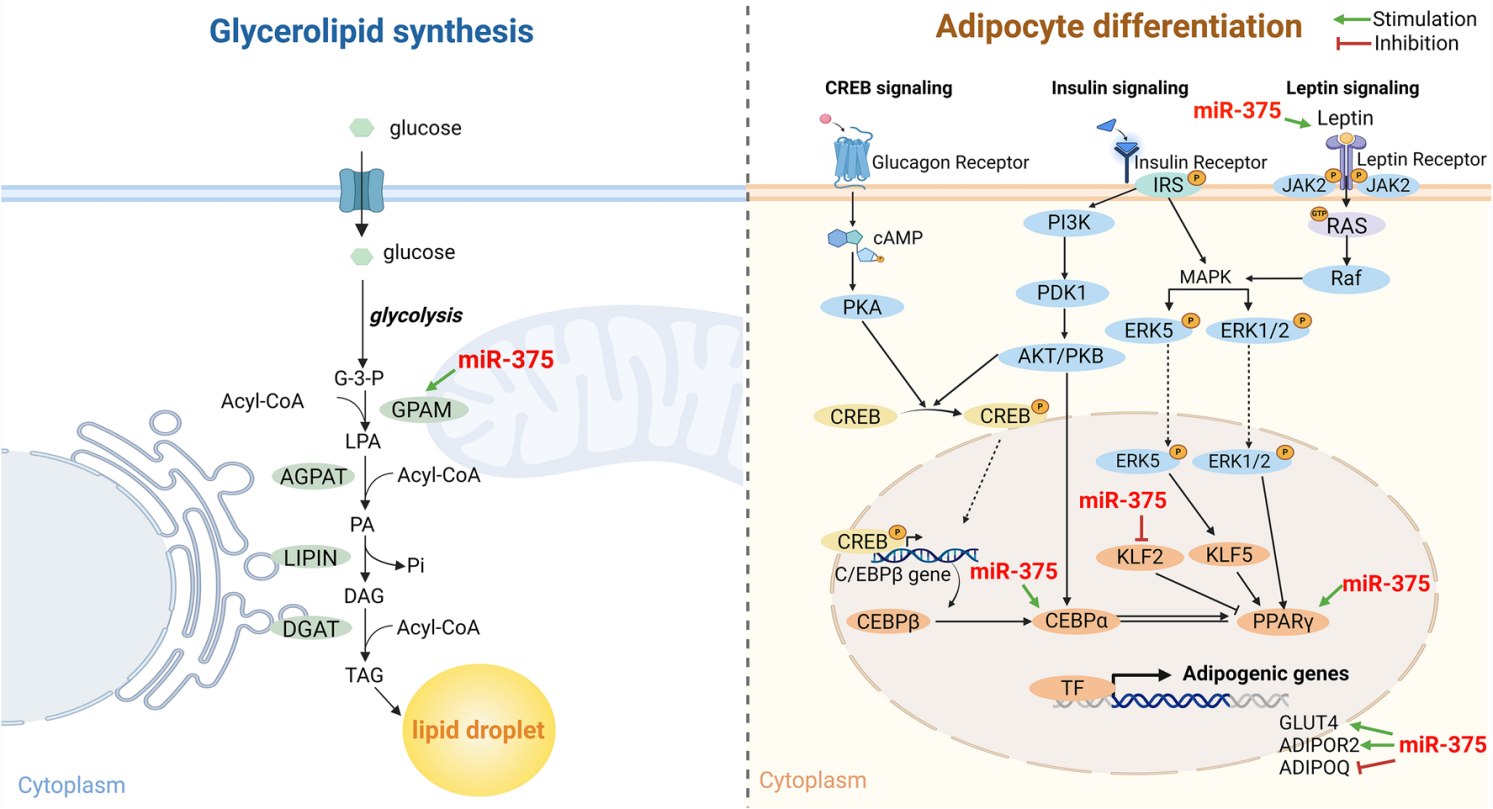
Gene regulatory function of miR-375 in 3T3-L1 cells. miR-375 upregulates *GPAM*, which plays a critical role in the synthesis of triglycerides and phospholipids in glycerolipid synthesis and downregulates the leptin gene, which suppresses lipogenesis and promotes lipolysis. The genes *CEBPα* and *PPARγ*, which encode transcription factors that differentiate adipocytes through adipogenesis, *GLUT4*, which encodes a glucose transporter involved in insulin-stimulated glucose uptake, and ADIPOR2, which encodes a receptor for adiponectin that promotes fatty acid degradation, are upregulated. In contrast, expression of the *ADIPOQ* gene is downregulated by miR-375. Additionally, miR-375 also downregulates the *KLF2* gene, which negatively regulates lipid metabolism. Taken together, these results indicate that miR-375 is a gene regulator that improves adipocyte differentiation and lipid accumulation.

Recent studies have revealed the critical roles of miRNAs in the proliferation, differentiation, and maturation of adipocytes. Studies on the regulation of fat metabolism-related genes by miRNA have emphasized the importance of selecting miRNAs that can improve bovine meat quality. IMF content is determined by various lipid metabolic processes such as adipogenesis, lipogenesis, glycerolipid synthesis, and lipolysis^2^. Genes involved in lipid metabolism include *C/EBPα*, *PPARγ*, and *KLF2* for adipogenesis; *LEP*, *ADIPOQ*, and *ADIPOR2* for fatty acid oxidation; *FASN* and *FABP4* for lipogenesis; and *GPAM*, *AGPAT* for glycerolipid synthesis. Although several studies have studied the genes involved in lipid metabolism, recent studies have focused on identifying the regulatory factors that control these fat metabolism-related genes. Gene expression is regulated through DNA methylation, histone modifications, and various RNA-mediated processes at the transcriptional level^41^. Protein-coding RNA comprise a minor part of the transcriptome, with the majority being ncRNA that play a significant role in post-transcriptional regulation. The roles of miRNAs (which are a type of ncRNA) in lipid metabolism, adipogenesis, and fat development have been extensively studied in human and mouse cell lines. Current studies are aiming to identify the miRNAs capable of regulating IMF in beef cattle^42^. bta-miR-143 enhances bovine intramuscular preadipocyte differentiation by regulating *C/EBPα* and *FABP4*^43^. bta-miR-149-5p down-regulates the adipogenic marker genes *PPARγ*, *C/EBPα*, *ELOVL6*, *LPL*, *SREBP-1*, *ACLY*, *PLN2*, *PLN1*, *ACCα*, *CRTC2*, *CRTC1*, and *AKT*, thereby inhibiting bovine adipocyte proliferation and differentiation^44^. bta-miR-378 promotes differentiation by increasing *PPARγ* and *C/EBPβ* mRNA expression levels and decreasing pref-1, adiponectin, and CaMKK2 expression in bovine adipocytes^45^. miR-375 is a multifunctional regulator of various cellular pathways and was initially studied for its function in pancreatic endocrine cells. It was identified as an evolutionarily conserved islet-specific miRNA^46^. Recent studies have shown that miR-375 is involved in various biological regulatory pathways and possess regulatory functions in immunity, inflammation, development, viral infections, and cancer^47^. Overexpression of miR-375 in 3T3-L1 cells enhanced adipocyte differentiation by reducing ERK1/2 phosphorylation and increased the mRNA expression of key differentiation transcription factors (CEBPα and PPARγ) and aP2, which results in triglyceride accumulation^48^. Seeliger et al*.*^49^ demonstrated that miR-375 knockdown during visceral preadipocyte differentiation upregulated the adipogenesis marker genes *ADIPOQ* and *ADIPOR2*. Gezginci-Oktayoglu et al*.*^50^ reported that reducing miR-375 expression improved Erk1 expression in ARIP cells, and suppression of miR-375 suppressed the expression of the adipogenesis markers PPAR2, LPL, GLUT4, and leptin. Kraus et al*.*^51^ showed that the suppression of miR-375 decreased human preadipocyte differentiation. Additionally, its inhibition increased ADIPOR2 expression, indicating its role as a regulator of adipogenic differentiation. Ouaamri et al.^52^ reported that miR-375 directly targets the *PDK1* gene and reduced PDK1 expression in pancreatic beta cells (Table 2). According to the results reported by Li et al.^53^, the expression of miR-375 is affected to the gene expression level in bovine intramuscular fat. Although the adipogenic effects of miR-375 have been well studied, its effects on *GPAM* remain unclear. This study aimed to investigate the regulatory role of miR-375 on the *GPAM* gene and its adipogenic effects on SNPs within the 3ʹ UTR of *GPAM* mRNA. Due to the absence of bovine adipose cell lines, the 3T3-L1 cell line was utilized to induce lipid accumulation in adipocytes. This study newly identified the function of miR-375 in up-regulating the *GPAM* gene in 3T3-L1 cells. Additionally, the study demonstrates that miR-375 plays a pivotal role in adipocytes by modulating several genes associated with lipid metabolism, as depicted in Fig. 5. Furthermore, miR-375 promotes lipid accumulation in adipocytes, indicating its crucial role in the storage of fat in adipocytes. Future studies should focus on elucidating the role of miRNAs in the regulation of lipid metabolism, specifically through targeting SNPs in Hanwoo adipocytes. In conclusion, miR-375, which is a multiple gene regulator targeting lipid metabolism-related genes, is not only an essential factor for improving lipid accumulation in adipocytes, but could also be used as a biomarker of lipid metabolism.

## Methods

### Cell lines

HEK293T and 3T3-L1 cell lines were provided by Chonnam National University, and the cells were cultured according to the protocol recommended by the ATCC (CL-173, CRL-3216).

### Construction of recombinant DNA with two *GPAM* 3ʹ UTR SNPs

Genomic DNAs was extracted from Hanwoo longissimus dorsi muscle samples using an AccuPrep Genomic DNA Extraction Kit (Bioneer, Daejeon, Korea). The NCBI Primer-BLAST program was used to design primers to amplify three candidate SNPs in the *GPAM* 3ʹ UTR. Each primer contained a restriction site for XhoI (forward primer only) and SalI (reverse primer only). A 15-bp sequence homologous to the pmirGLO vector sequence was used for performing homologous recombination using the seamless cloning method, which has a high ligation efficiency than that of the classical cloning method. Two different SNPs in the *GPAM* 3ʹ UTR were amplified using PCR with primers that were designed to have 15 homologous bases with the vector. PCR was performed using HotStart Taq DNA Polymerase (Bioneer, Daejeon, Korea) by following the manufacturer’s instructions. The amplified SNPs were inserted into the XhoI- and SalI- (New England Biolabs, Beverly, Massachusetts, USA) digested pmirGLO vector (Promega, Madison, Wisconsin, USA) backbone using a Gibson Assembly Cloning Kit (New England Biolabs, Beverly, Massachusetts, USA). The presence of two different SNPs (A-A-C and G-G-T) in the 3ʹ UTR of the *GPAM* gene in recombinant DNA was confirmed through sequencing, and the recombinant DNA was subsequently used for luciferase assays.

### HEK293T cell culture and miRNA transfection

Dual-luciferase reporter assays were performed in HEK293T cells that were cultured in Dulbecco’s modified Eagle’s medium/F-12 (Welgene, Seoul, Korea) containing 10% fetal bovine serum (Welgene, Seoul, Korea) and 1% penicillin–streptomycin (Welgene, Seoul, Korea) in a humified incubator at 5% CO_2_ and 37 °C. The HEK293T cells were plated in 96-well plates for transfection. miRNA mimics are chemically synthesized double-stranded RNA molecules that mimic mature miRNA duplexes. When cells reached 70% confluence, miRNA mimics (Bioneer, Daejeon, Korea) and recombinant DNA were co-transfected into the HEK293T cells using Lipofectamine 3000 (Invitrogen, Carlsbad, California, USA). The HEK293T cells were harvested 48 h after transfection, and luciferase activity was measured.

### Dual-luciferase reporter assay

The wild-type (A-A-C) and mutant (G-G-T) *GPAM* 3ʹ UTRs were cloned into the pmirGLO vector (Promega, Madison, Wisconsin, USA), which is a dual-luciferase reporter gene vector. HEK293T cells were co-transfected with miRNA mimics (50 nM) or negative control mimics (50 nM) and wild-type or mutant recombinant DNA using Lipofectamine 3000. Luciferase activity of the cells was measured using a Dual-Luciferase Reporter Assay System (Promega, Madison, Wisconsin, USA) 48 h after transfection. Relative luciferase activity was normalized to that of firefly luciferase. Renilla and firefly luciferase activities were measured using a BioTek Synergy HTX Multi-Mode Microplate Reader (BioTek Instruments, Vermont, USA).

### MiRNA conservation analysis

To determine whether the seed regions of bta-miR-2418, bta-miR-2479, bta-miR-375, and bta-miR-2468 were conserved across species, their sequences were downloaded from miRBase v.22.1 and MirGeneDB 2.1. Subsequently, miRNA sequences were analyzed using miRBase to confirm conservation across species. The seed regions of the miRNAs were confirmed and conserved miRNAs were selected by confirming that they matched the seed sequences of other species. Additionally, to confirm the homology between the bovine and mouse *GPAM* genes, transcript and protein accession numbers were obtained from NCBI (3ʹUTR: mouse, NC_000085.7; *Bos taurus*, NC_037353.1; mRNA: mouse, NM_008149.4; *Bos taurus*, XM_005225720.4; protein: mouse, NP_032175.2; *Bos taurus*, XP_005225777.1). The degree of homology was confirmed using NCBI BLAST.

### Differentiation of 3T3-L1 cells and miRNA transfection

The 3T3-L1 cells were cultured in high glucose Dulbecco’s modified Eagle’s medium (Welgene, Seoul, Korea) containing 10% bovine calf serum (Welgene, Seoul, Korea) and 1% penicillin–streptomycin in a humified incubator at 5% CO_2_ and 37 °C. To induce differentiation in the 3T3-L1 cells, they were plated onto 24-well plates and cultured until they reached confluency. Then, the growth media was changed to a differentiation media containing 10% FBS, 3-isobutyl-1-methylxanthine (0.5 mM; Sigma-Aldrich, St Louis, MO, USA), dexamethasone (0.001 mM; Sigma-Aldrich, St Louis, MO, USA), pioglitazone (0.002 mM; Sigma-Aldrich, St Louis, MO, USA), and insulin (0.005 mg/mL; Sigma-Aldrich, St Louis, MO, USA). After treatment for 2 days with the differentiation medium, the medium was changed to an insulin medium containing 10% FBS and insulin (0.005 mg/mL) for another 2 days. The cells were cultured in high-glucose DMEM with 10% FBS and 1% penicillin–streptomycin, and the medium was changed every 2 days. To determine the effect of miRNAs on the expression of lipid metabolism-related genes in adipocytes, miR-375 mimics were transfected into 3T3-L1 cells using Lipofectamine 3000 every 3 days when the cells reached 70% confluency. The 3T3-L1 cells were cultured for up to 12 days after transfection, and qRT-PCR was performed every 2 days to analyze the expression levels of the lipid metabolism-related genes. The miRNA transfection efficiency in 3T3-L1 cells was confirmed using a Nikon Eclipse Ti2-U microscope (Nikon Instruments, New York, NY, USA), and the images were analyzed using Nikon Imaging Software (NIS)-Elements v.5.42.01, https://www.nis-elements.cz/en (Nikon Instruments).

### RNA isolation and quantitative real-time PCR

The 3T3-L1 cells were lysed using TRIzol Reagent (Invitrogen, Carlsbad, CA, USA) according to the manufacturer's instructions. Subsequently, 1-Bromo 3-chloropropane (Sigma Aldrich, Saint Louis, USA) was added for RNA isolation. Total RNA was then purified using the RNeasy Mini Kit (Qiagen, Valencia, CA, USA). cDNA was synthesized by performing reverse transcription with oligo dT primers and Superscript II reverse transcriptase (Invitrogen, Carlsbad, CA, USA). qPCR was performed using a SYBR Premix Ex Taq kit (TaKaRa Bio) on a CFX96 system (Bio-Rad, Hercules, CA, USA). Relative expression levels of mRNAs were calculated using the 2^−∆∆Ct^ method. The primer sequences used for qRT-PCR are listed in Supplementary Table S1.

### Oil Red O staining and triglycerides assay

To measure cell lipid droplets, 3T3-L1 adipocytes were washed thrice with PBS and fixed in 10% formalin (FUJIFILM Wako Pure Chemical Corporation, Japan) for 10 min at 24 °C. Then, 10% formalin was discarded, and fresh 10% formalin was added. The cells were incubated for 1 h at 24 °C and then washed with tertiary distilled water. The cells were incubated with 60% isopropanol and allowed to stand for 5 min at room temperature. After drying, Oil Red O solution (500 µL) was added to each well and incubated at room temperature for 20 min. The cells were washed thrice with tertiary distilled water and photographed using a Nikon Eclipse Ti2-U microscope (Nikon Instruments, NY, USA). The images were analyzed using the NIS-Elements software (Nikon Instruments, USA). For the triglyceride assays, the Oil Red O stain was extracted with 100% isopropanol, and the optical density (OD) was measured at 510 nm.

### Statistical analysis

Data are expressed as mean ± standard deviation (SD). To analyze relative luciferase activity data, the statistical differences between the two groups were determined using the Student's t-test^54^. For the statistical analysis of qRT-PCR data, comparisons between multiple groups were performed using the two-way analysis of variance (ANOVA)^55^. Differences were considered statistically significant at p < 0.05.

### Supplementary Information

Supplementary Information.

## Data Availability

Sequence data is available in the GenBank repository, PP458229-PP458232. All the data generated in this study have been included in the manuscript. For further access, please contact the corresponding author, Yoonseok Lee.
