# Supplementary material for: SNPs in microRNA seed region and impact of miR-375 in concurrent regulation of multiple lipid accumulation-related genes
Source: Sci Rep. 2024 May 13;14:10924. doi: 10.1038/s41598-024-61673-4 (PMC11091151; doi:10.1038/s41598-024-61673-4)
Supplement: Supplementary file 1 — Supplementary Information. [file 41598_2024_61673_MOESM1_ESM.pdf]

Supplementary information

SNPs in microRNA Seed Region and Impact of miR-375 in Concurrent Regulation of Multiple Lipid Accumulation-related Genes

Jiyeon Lee [1], Inpyo Hong[1], Chanwoo Lee [2], Daehyun Kim[3], Sunghak Kim [3]*, Yoonseok Lee[1,4]*

[1] School of Biotechnology, Hankyong National University, Gyeonggi-do, Anseong, Korea
[2] Nuonbio Inc., 906, A, 302 Galmachi-ro, Jungwon-gu, Seongnam-si, Republic of Korea
[3] Department of Animal Science, Chonnam National University, Gwangju, Korea
[4] Center for Genetic Information, Hankyong National University, Gyeonggi-do, Anseong, Korea

* Corresponding author: Yoonseok Lee (yoonseok95@hknu.ac.kr)
Sunghak Kim (sunghakkim@chonnam.ac.kr)

Gene	Forward primer (5'- 3')	Reverse primer (5'- 3')	Reference
C/EBPα	CGCAAGAGCCGAGATAAAGC	CGGTCATTGTCACTGGTCAACT	57
PPARγ	GAAAGACAACGGACAAATCACC	GGGGGTGATATGTTTGAACTTG	58
KLF2	CTGCGTACACACACAGGTGA	GTGGCACTGAAAGGGTCTGT	59
PDK1	CACCACGCGGACAAAGG	GCCCAGCGTGACGTGAA	60
FABP4	CATGGCCAAGCCCAACAT	CGCCCAGTTTGAAGGAAATC	57
GLUT4	AGAGTCTAAAGCGCCT	CCGAGACCAACGTGAA	61
ADIPOQ	TGTTCCTCTTAATCCTGCCCA	CCAACCTGCACAAGTTCCCTT	62
ADIPOR2	CACAACCTTGCTTCA	ATACTGAGGGGTGGC	63
LEP	GAGACCCCTGTGTCGGTTC	CTGCGTGTGTGAAATGTCATTG	64
GPAM	GAGGAGTCTTCAGTGACAGTTG	CAGTCCTCACTGGTGTGTTT	65
β-Actin	TGTCCACCTTCCAGCAGATGT	AGCTCAGTAACAGTCCGCCTAGA	66

Supplementary Table S1. Primer sequences used for quantitative real-time PCR

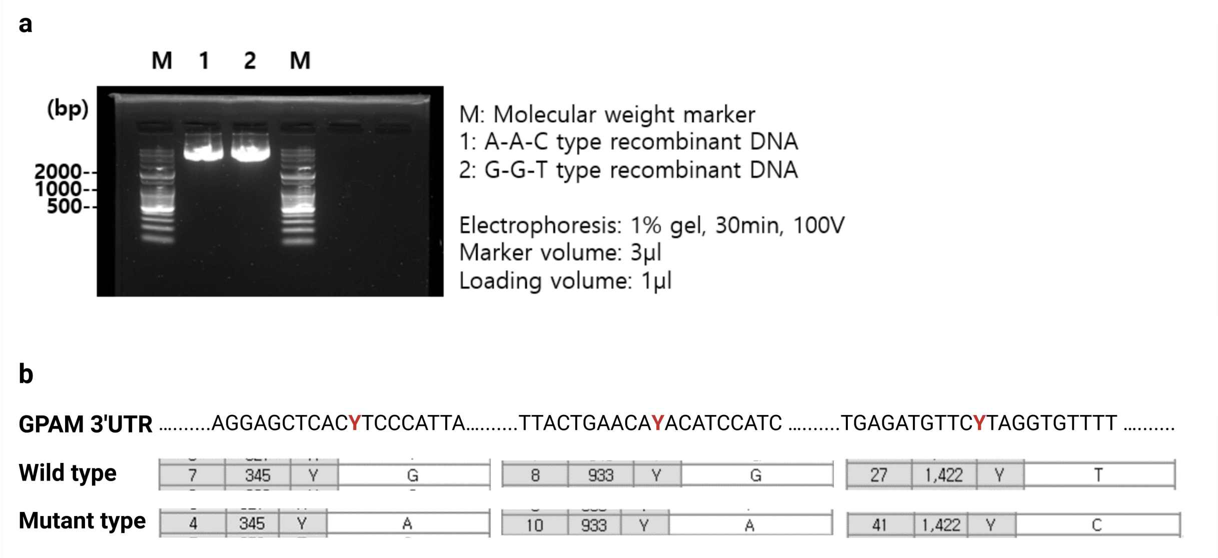

Supplementary Figure S1. Construction of recombinant DNA with two GPAM 3' UTR SNPs. (a) Electrophoresis result of recombinant DNA constructed by Gibson assembly. The recombinant DNAs contained two types of SNPs combination of GPAM 3'UTR. **(b)** The presence of two different SNPs (A-A-C and G-G-T) in the 3'UTR of the GPAM gene in the recombinant DNA was confirmed by sequencing.

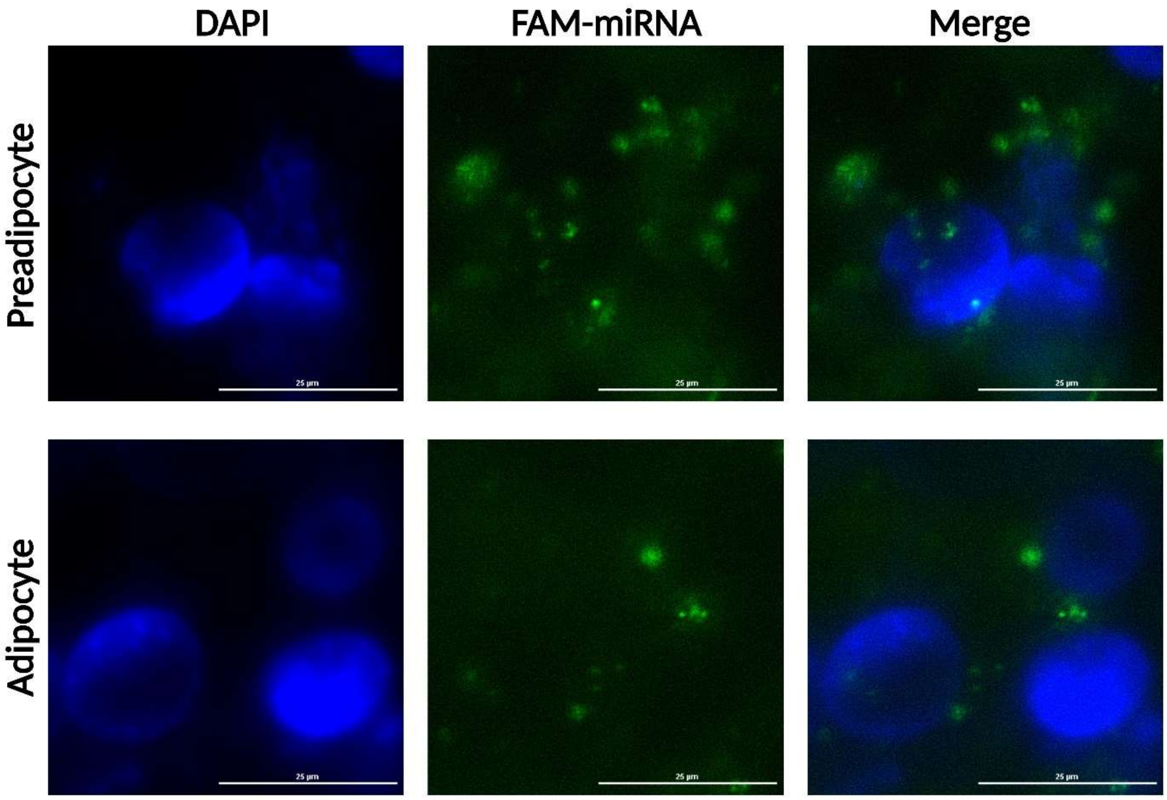

Supplementary Figure S2. Transfection efficiency of FAM-labeled bta-miR-375 mimic in 3T3-L1 cells (scale bar, 25 µm).
